# Association of lymphocyte subsets with the efficacy and prognosis of PD‑1 inhibitor therapy in advanced gastric cancer: results from a monocentric retrospective study

**DOI:** 10.1186/s12876-024-03168-0

**Published:** 2024-03-15

**Authors:** Xinyan Wang, Xiaoling Liu, Huwei Dai, Junmei Jia

**Affiliations:** 1 The First Clinical Medical College of Shanxi Medical University, No.56, Xinjian South Road, Yingze District, Taiyuan, Shanxi 030001 People’s Republic of China; 2 Department of Oncology, The First Hospital of Shanxi Medical University, No.85, Jiefang South Road, Yingze District, Taiyuan, Shanxi 030001 People’s Republic of China; 3 Department of Special Medical, Shanxi Province Cancer Hospital, Taiyuan, Shanxi 030013 China; 4 The Second Clinical Medical College of Shanxi Medical University, Taiyuan, Shanxi 030001 China

**Keywords:** Advanced gastric cancer, Immunotherapy, Biomarkers, Peripheral blood lymphocyte subsets

## Abstract

**Purpose:**

This retrospective study aimed to investigate the changes in peripheral blood lymphocyte subsets before and after immunotherapy in patients with advanced gastric cancer and their relationship n with the therapeutic efficacy and clinical prognosis.

**Methods:**

Peripheral blood lymphocyte subsets, including CD4 + T cells, CD8 + T cells, CD4+/CD8 + ratio, NK cells, Treg cells, and B cells, were collected from 195 patients with advanced gastric cancer who were admitted to the First Hospital of Shanxi Medical University with immunotherapy from January 2020 to October 2021, at the time of diagnosis of advanced gastric cancer, before immunotherapy and after 3 cycles of immunotherapy. T-tests were used to examine the factors influencing the patients’ peripheral blood lymphocyte subsets and the changes after immunotherapy. To examine the relationship between lymphocyte subsets and treatment outcomes, ROC curves were plotted using a logistic regression. Kaplan–Meier curve was drawn, and the Log Rank test was carried out to compare the differences in PFS between the different groups. Cox proportional hazards regression model was used to analyze the factors affecting PFS after calibration of other variables.

**Results:**

The proportion of peripheral blood lymphocyte subsets in patients with advanced gastric cancer was affected by age and PD-L1 level. Compared to the baseline, the treatment effective group had higher proportions of CD4 + T cells, a higher CD4+/CD8 + ratio, NK cells and Treg cells, and lower proportions of CD8 + T cells and B cells in the peripheral blood after three cycles of immunotherapy. In the treatment-naive group, there were no significant differences in the lymphocyte subsets. With cut-off values of 30.60% and 18.00%, baseline CD4 + T cell and NK cell ratios were independent predictors of immunotherapy efficacy and PFS. Treg cell ratio, gender, PD-L1 levels, and MMR status all predicted PFS independently.

**Conclusion:**

The proportion of peripheral blood lymphocyte subsets was modified in patients who responded to PD-1 inhibitors. Different lymphocyte subpopulation levels can be used as biomarkers to predict immunotherapy efficacy and clinical prognosis in patients with advanced gastric cancer.

**Supplementary Information:**

The online version contains supplementary material available at 10.1186/s12876-024-03168-0.

## Introduction

Gastric cancer is a serious threat to human health, with the fifth highest incidence and fourth highest mortality rate among malignant tumors worldwide [1]. This has put a significant strain on global healthcare [2]. Immune checkpoint inhibitors have changed the landscape of medical treatment for advanced gastric cancer, and have become the mainstay of medical treatment for advanced gastric cancer, along with targeted therapy and chemotherapy. A number of clinical trials have shown that immune checkpoint inhibitors are effective and safe in gastric cancer, however, in clinical practice, some patients have difficulty benefiting from immunotherapy, and the selection of appropriate biomarkers to maximize patient benefit is an urgent issue.

Anti-PD-1 and its combination therapies are the most commonly used immunotherapies. The mechanism mainly involves blocking the binding of PD-1 and PD-L1, promoting the activation and proliferation of T cells in the tumor, enhancing the tumor-killing effect of T cells, and improving the body’s anti-tumor immunity [3]. Commonly used clinical efficacy biomarkers include PD-L1, TMB and MSI [4]. However, these biomarkers require tissue specimens that are invasive, expensive, and cannot be monitored dynamically. The discovery of biomarkers in peripheral blood that may be related to immune checkpoint inhibitors (ICI) treatment response may provide a simple non-invasive method for monitoring patient treatment.

The immune system is the engine that drives immunotherapy and plays an important role in tumor surveillance and host protection [5]. Tumor infiltrating lymphocytes (Tils), an important component of the immune system, have been shown to correlate with the prognosis of gastric cancer [6]. On the other hand, the detection of Tils necessitates pathological sections with high tumor tissue specificity, and the location of sampling has a significant impact on the results. A wide range of cytokines and lymphocytes migrate from the peripheral blood into the tumor microenvironment, primarily via body circulation [7]. Studies addressing the relationship between peripheral blood CD4 + T cells, CD8 + T cells, Treg cells, and B cells and the effectiveness and progression-free survival of immunotherapy for advanced gastric cancer are lacking.

In this study, we investigated the relationship between peripheral blood lymphocyte subsets and the predictive role and efficacy of PD-1 inhibitor therapy in patients with advanced gastric cancer by analyzing the changes in peripheral blood lymphocyte subsets before and after receiving PD-1 inhibitor therapy. Additionally, we analyzed the relationship between peripheral blood lymphocyte subsets and the clinicopathological characteristics in patients with advanced gastric cancer.

## Patients and methods

### Study design

Data were collected from 266 patients with advanced gastric cancer treated with PD-1 inhibitors who were hospitalized at the Department of Oncology at The First Hospital of Shanxi Medical University between January 2020 and October 2021. Pembrolizumab, nivolumab, tislelizumab, toripalimab, camrelizumab and sintilimab were the different PD-1 inhibitors. Inclusion criteria: (1) clinical stage IV, her-2 negative gastric adenocarcinoma confirmed by imaging and histopathological examination; (2) receiving at least three cycles of PD-1 inhibitor monotherapy, PD-1 inhibitor combined with chemotherapy, or PD-1 inhibitor combined with anti-angiogenic drugs at the First Hospital of Shanxi Medical University; (3) ECOG performance status between 0 and 2; (4) availability of complete lymphocyte subsets, PD-L1, mismatch repair (MMR) status and the follow-up data; exclusion criteria: (1) her-2-positive gastric adenocarcinoma; (2) suffering from other types of malignant tumors; (3) had acute infections, immunodeficiency, and autoimmune illnesses; (4) suffering from underlying cardiac, pulmonary, hepatic, and renal problems; (5) currently undergoing systemic hormone therapy. 195 of these patients met these requirements and were included in the trial. 27 patients were excluded because of insufficient data on lymphocyte subsets, 23 patients were excluded because of insufficient PD-L1 and MMR results, 6 patients were excluded because of severe underlying conditions, and 15 patients were excluded because of insufficient follow-up data.

All the participants gave an informed consent. This study was approved by the Ethical Committee of Te first Hospital of Shanxi Medical University (Ethics No. KYLL-2023-011).

### Data collection

Clinical and pathological data of patients were gathered, including diagnosis, gender, age, BMI, smoking history and drinking history, presence of extensive metastases at the time of the initial diagnosis of advanced gastric cancer, treatment regimen, and number of lines of therapy. Laboratory data included PD-L1 expression levels, MMR status, and peripheral blood lymphocyte subset test results (CD4 + T cells, CD8 + T cells, CD4/CD8 ratio, NK cells, Treg cells, and B cells) at the time of initial diagnosis of advanced gastric cancer, before immunotherapy, and after three cycles of immunotherapy. Peripheral blood lymphocyte counts for patients receiving first-line therapy were the same at the time of initial diagnosis of advanced gastric cancer and prior to immunotherapy. On the basis of computed tomography (CT), magnetic resonance imaging (MRI), or PET-CT, the effectiveness of immunotherapy is evaluated. According to the solid tumor response evaluation criteria (RECIST 1.1), short-term clinical efficacy was divided into four categories: complete response (CR), partial response (PR), stable disease (SD), and progressive disease (PD). Patients evaluated as PD were classified as the ineffective group, and those evaluated as CR, PR, and SD were classified into the effective group. Progression-free survival (PFS) is defined as the time from the start of treatment to imaging or clinical progression or death from any cause according to RECIST (RECIST-PFS). PD-L1 CPS ≥ 5 was defined as PD-L1 positive. And PD-L1 CPS<5 was defined as PD-L1 negative. Extensive metastasis is defined as having metastatic lesions more than or equal to 2.

### PD‑L1 immunohistochemistry assessment

Immunohistochemical staining was performed on the VENTANA BenchMark Ultra platform using the kit accompanying the instrument. PD-L1 positive immunoreactive staining was localized to the cell membrane and assessed using the combined positive score (CPS), which is 100 times of the ratio of the total number of immunoreactively stained tumor cells, lymphocytes and macrophages in the 20x objective field to the total number of tumor cells in the field. The materials and procedures are provided in additional file 1.

### Mismatch repair status immunohistochemistry assessment

MMR staining was performed by immunohistochemical staining on the Leica BOND III platform using the platform’s kit. Absence of any one or more of the four MMR proteins (MLH1, MSH2, MSH6 and PMS2) was defined as mismatch repair deficient (dMMR), and all positives were defined as mismatch repair proficient (pMMR). The materials and procedures are provided in additional file 2.

### Blood collection and fow cytometry

The proportion of lymphocyte subsets in the peripheral blood was measured using a BD FAS-Canto II flow cytometer and flow antibodies. According to the flow cytometry testing platform at the First Hospital of Shanxi Medical University, the normal reference ranges for the above parameters are: CD4 + T cells (30–50%), CD8 + T cells (20–35%), CD4+/CD8 + ratio (1–2), NK cells (20–35%), Treg cells (3–7%) and B cells (5.6–16%). The materials and procedures are provided in additional file 3.

### Statistical methods

SPSS version 23.0 statistical software was employed for the analysis. Number of cases(%) were used to describe qualitative data. Quantitative data was expressed as mean ± standard deviation. The paired t-test was used to examine changes in lymphocyte subsets before and after immunotherapy. The t-test was used to examine differences in lymphocyte subsets in various groups with various clinicopathological characteristics at the time of the initial diagnosis of advanced gastric cancer. The variations in lymphocyte subsets, PD-L1 expression levels, and MMR status in various efficacy groups were compared using the t-test and χ^2^ test. The relevance of lymphocyte subsets in predicting the efficiency of immunotherapy in advanced gastric cancer was further clarified through ROC curves and logistic regression analysis to discover independent predictors of immunotherapy efficacy. Kaplan–Meier curve was drawn, and the log-rank test was performed to compare the differences in PFS between the different groups. Cox proportional hazard regression model was used to analyze the risk of poor prognosis among different groups after other variables were calibrated. Generally, results with p values of ≤ 0.05 were considered to be statistically significant for all analyses.

## Results

### Patient characteristics

A total of 195 patients with advanced gastric cancer were enrolled in this study, of whom 9 were dMMR, 186 were pMMR, 12 had immunotherapy alone, 173 had immunotherapy in combination with chemotherapy, and 10 had immunotherapy combined with anti-angiogenic targeted treatment. These patients were between 46 and 84 years old. 90 patients had PD-L1 CPS ≥ 5 and 105 patients had PD-L1 CPS<5. The clinical and pathological characteristics of patients are reported in Table 1.

**Table 1:** Clinical and pathological characteristics

Characteristics		*N*	%
Age			
<65		117	60.00
≥ 65		78	40.00
Gender			
Female		54	27.69
Male		141	72.31
BMI			
BMI<18.5 kg/m2		35	17.95
BMI ≥ 18.5 kg/m2		160	82.05
Smoking history			
No		106	54.36
Yes		89	45.64
Drinking history			
No		141	72.31
Yes		54	27.69
Extensive metastasis			
No		78	40.00
Yes		117	60.00
PD-L1 expression			
CPS<5		105	53.85
CPS ≥ 5		90	46.15
Mismatch repair status			
pMMR		186	95.38
dMMR		9	4.62
Treatment line			
First line		53	27.18
Second line and beyond		142	72.82
Treatment model			
ICIs alone		12	6.15
Chemotherapy combined with ICIs		173	88.72
Anti-angiogenesis drugs combined with ICIs		10	5.13
Tumor response			
Complete response		2	1.03
Partial response		53	27.18
Stable disease		89	45.64
Progressive disease		51	26.15

### Factors influencing lymphocyte subset in patients with advanced gastric cancer

Information on lymphocyte subsets in patients with advanced gastric cancer at the time of initial diagnosis of advanced gastric cancer was collected and we analyzed the impact of clinicopathological features on the different lymphocyte subsets by univariate analysis (Table 2). Patients aged <65 years had a lower proportion of NK cells than those aged ≥ 65 (*P* = 0.017). Patients with PD-L1 CPS<5 exhibited lower levels of CD4+/CD8 + ratio and a larger proportion of CD8 + T cells (*P* = 0.015) compared to patients with PD-L1 CPS ≥ 5. The lymphocyte subsets were unaffected by gender, smoking history, drinking history, BMI, MMR status, or the presence of extensive metastases. The relationship between peripheral lymphocyte subsets and clinicopathological features in patients with advanced gastric cancer is shown in Table 2.

**Table 2:** The impact of clinical and pathological characteristics on lymphocyte subsets

	CD4 + T	*P*	CD8 + T	*P*	CD4+/CD8+	*P*	NK	*P*	Treg	*P*	B	*P*
Age												
<65	37.68 ± 9.65	0.088	30.98 ± 10.63	0.399	1.42 ± 0.71	0.984	19.95 ± 10.33	0.017	5.84 ± 2.42	0.369	7.52 ± 3.46	0.207
≥ 65	35.28 ± 9.51		29.64 ± 11.12		1.42 ± 0.82		23.38 ± 11.01		6.26 ± 4.07		6.89 ± 3.35	
Gender												
Female	38.69 ± 11.01	0.078	29.55 ± 11.03	0.480	1.51 ± 0.72	0.299	20.35 ± 11.94	0.524	5.81 ± 2.17	0.605	7.42 ± 3.21	0.709
Male	35.97 ± 8.99		30.78 ± 10.76		1.39 ± 0.77		21.44 ± 10.27		6.08 ± 3.49		7.21 ± 3.51	
BMI												
BMI<18.5 kg/m2	37.99 ± 9.15	0.393	28.08 ± 9.91	0.154	1.62 ± 0.92	0.088	21.04 ± 10.31	0.949	6.31 ± 5.37	0.529	7.54 ± 3.28	0.597
BMI ≥ 18.5 kg/m2	36.44 ± 9.75		30.96 ± 10.97		1.38 ± 0.71		21.16 ± 10.86		5.94 ± 2.48		7.21 ± 3.46	
Smoking history												
No	35.74 ± 9.99	0.123	31.47 ± 11.08	0.146	1.35 ± 0.77	0.140	20.76 ± 11.33	0.588	5.99 ± 3.94	0.963	7.02 ± 3.67	0.271
Yes	37.88 ± 9.13		29.21 ± 10.43		1.51 ± 0.74		21.60 ± 10.03		6.02 ± 1.93		7.57 ± 3.11	
Drinking history												
No	36.43 ± 9.80	0.494	31.03 ± 11.54	0.233	1.40 ± 0.76	0.548	21.16 ± 11.23	0.961	5.89 ± 3.54	0.408	7.09 ± 3.47	0.245
Yes	37.49 ± 9.26		28.91 ± 8.59		1.47 ± 0.76		21.08 ± 9.43		6.31 ± 1.92		7.73 ± 3.28	
Extensive metastasis												
No	36.87 ± 10.39	0.856	30.57 ± 11.24	0.893	1.45 ± 0.84	0.620	20.73 ± 10.84	0.663	6.23 ± 3.94	0.423	7.37 ± 3.58	0.730
Yes	36.62 ± 9.15		30.35 ± 10.58		1.40 ± 0.70		21.42 ± 10.71		5.85 ± 2.56		7.20 ± 3.33	
PD-L1 expression												
CPS<5	35.49 ± 8.96	0.055	32.17 ± 11.56	0.015	1.30 ± 0.71	0.024	20.89 ± 10.50	0.728	6.00 ± 3.61	0.977	7.02 ± 3.03	0.277
CPS ≥ 5	38.15 ± 10.24		28.42 ± 9.56		1.55 ± 0.78		21.43 ± 11.06		6.01 ± 2.61		7.56 ± 3.83	
Mismatch repair status												
pMMR	36.89 ± 9.68	0.269	30.54 ± 10.89	0.566	1.43 ± 0.76	0.709	21.03 ± 10.90	0.519	5.93 ± 3.18	0.165	7.24 ± 3.44	0.533
dMMR	33.24 ± 8.49		28.41 ± 9.62		1.33 ± 0.64		23.40 ± 6.29		7.44 ± 3.10		7.97 ± 3.16	

### Comparison of peripheral blood lymphocyte subsets in patients in different efficacy groups

**Fig. 1:**
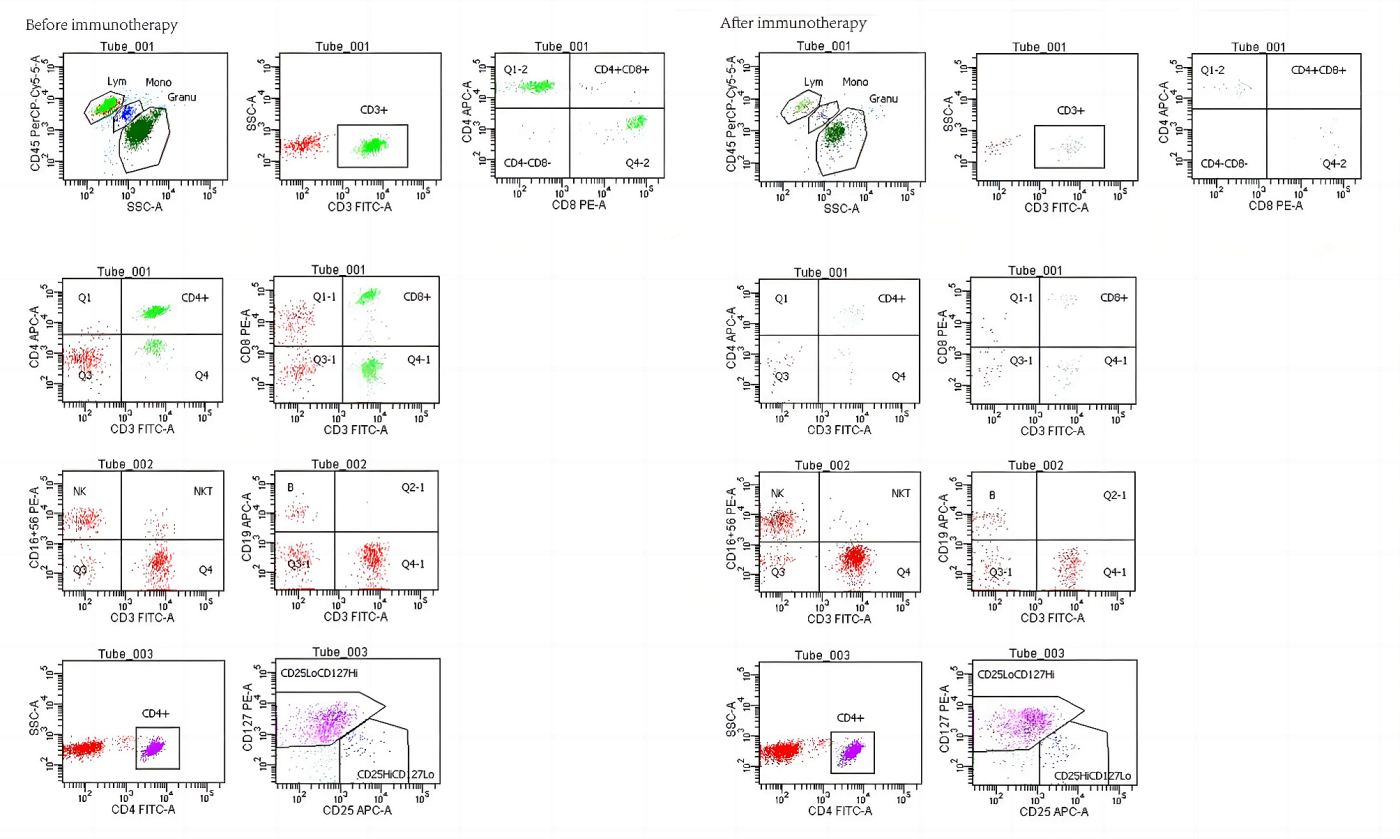
The fow image of a typical patient before and after immunotherapy

A total of 195 patients were included in this study and the efficacy of immunotherapy was evaluated after three cycles, divided into 144 in the effective group (CR 2 PR 53 SD 89) and 51 in the ineffective group, with a treatment efficiency of 76.4%. The proportion of CD4 + T cells (*P* = 0.031), NK cells (*P* = 0.032) Treg cells (*P* = 0.024) and the ratio of CD4+/CD8+ (*P* = 0.002) increased significantly in the effective group. The proportion of B cells (*P* = 0.018) and CD8 + T cells (*P* = 0.030) decreased after treatment compared to the baseline. There were no significant changes in the lymphocyte subsets in the ineffective group. Figure 1 shows a typical flow chart of the peripheral lymphocyte subsets in patients before and after immunotherapy. A comparison of the peripheral blood lymphocyte subsets in patients in the different efficacy groups is shown in Table 3.

**Table 3:** Comparison of the different lymphocyte subsets before and after treatment in the immunotherapy effective and ineffective group

	Effective group			Ineffective group		
	Before	After	*P*	Before	After	*P*
CD4+	37.04 ± 7.36	38.82 ± 7.36	0.031	33.40 ± 7.73	34.92 ± 9.92	0.222
CD8+	30.46 ± 8.48	28.14 ± 9.45	0.030	37.14 ± 14.30	34.22 ± 12.64	0.252
CD4+/CD8+	1.33 ± 0.53	1.56 ± 0.65	0.002	1.06 ± 0.55	1.25 ± 0.76	0.064
NK	21.96 ± 9.79	24.45 ± 9.99	0.032	17.44 ± 10.31	19.06 ± 10.57	0.389
Treg	6.80 ± 2.47	7.39 ± 1.85	0.024	6.01 ± 2.27	5.45 ± 1.83	0.303
B	6.11 ± 3.22	5.25 ± 2.83	0.018	5.30 ± 3.29	6.04 ± 4.79	0.362

### Correlation of baseline lymphocyte subsets with short-term efficacy

**Fig. 2:**
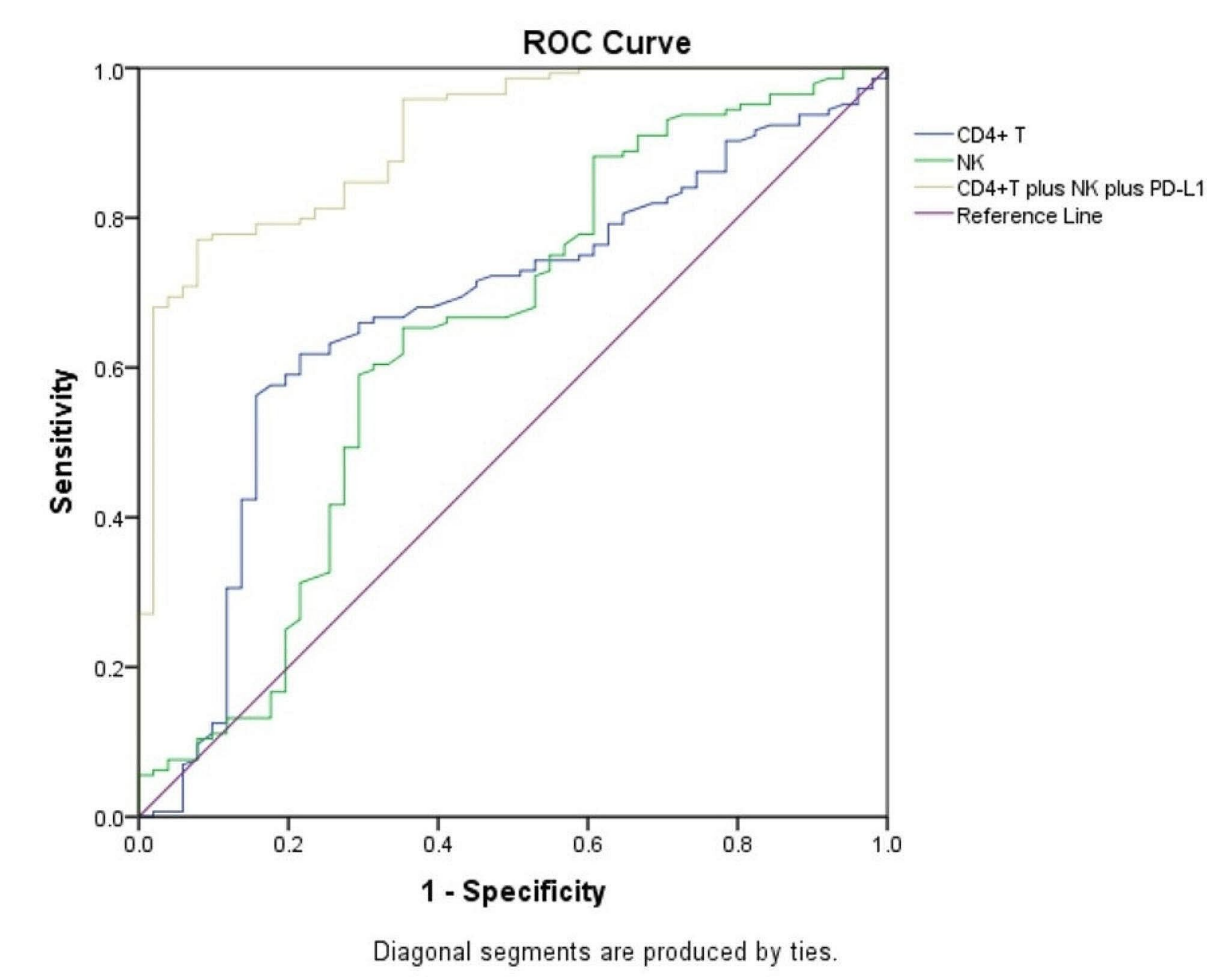
The ROC curve of CD4 + T cells, NK cells and CD4 + T cells, NK cells combined with PD-L1 before immunotherapy

Differences in baseline lymphocyte subsets between the 144 in effective group and the 51 in ineffective group were compared and showed that CD4 + T cells (*p* = 0.002), NK cells (*p* = 0.003), and Treg cells (*p* = 0.046) were higher in effective group and CD8 + T cells (*p* = 0.002) were lower than those in the ineffective group. The differences in PD-L1 expression, MMR status, gender, age, BMI, smoking history, drinking history and extensive metastasis between the two groups were compared using the chi-square test, which showed a higher proportion of PD-L1 positive patients in the effective group (*P*<0.001), while the remaining indicators did not differ between the two groups (*P* > 0.05). Including CD4 + T cells, NK cells, Treg cells, CD8 + T cells and PD-L1 as variables in the logistic regression model, the results showed that PD-L1 (OR: 0.005, 95% CI: 0.001–0.044, *P*<0.001), CD4 + T cells (OR: 0.870, 95% CI: 0.790–0.959, *P* = 0.005), NK cells (OR: 0.887, 95% CI: 0.815–0.965, *P* = 0.005), and Treg cells (OR: 0.712, 95% CI: 0.567–0.895, *P* = 0.004) were factors influencing the short-term efficacy of immunotherapy. The proportion of CD8 + T cells did not correlate with short-term patient outcomes (*P*>0.05). Additionally, our ROC curves demonstrated that the predictive efficacy of CD4 + T cells and NK cells was statistically significant (*P*<0.05), whereas that of Treg cells was not statistically significant (*P* = 0.078). The AUCs of CD4 + T cells and NK cells were 0.672 and 0.639 (Fig. 2). The maximum Youden index based on ROC curves was 30.60 (sensitivity = 0.563, specificity = 0.843) and 18.00 (sensitivity = 0.653, specificity = 0.647) for CD4 + T cells and NK cells. And the combination of CD4 + T cells, NK cells and PD-L1 had better diagnostic power than individual predictors, with an AUC of 0.909 (*P* < 0.001).

In addition we analyzed patients based on MMR status and treatment regimen. In the pMMR group, the treatment-effective patient group had more PD-L1-positive patients and had higher levels of CD4 + T cells (*P* = 0.001), NK cells (*P* = 0.010), and lower levels of CD8 + T cells (*P* < 0.001), CD4 + T cells, CD8 + T cells, NK cells, and PD-L1 status of patients in the pMMR group were included as as variables in the logistic regression model. The results showed that PD-L1 (OR: 0.009, 95% CI: 0.001–0.073, *P* < 0.001), CD4 + T cells (OR: 0.870, 95% CI: 0.790–0.959, *P* = 0.006), NK cells (OR: 0.899, 95% CI: 0.835–0.969, *P* = 0.005) were factors that influenced the short-term outcome of patients. In the dMMR group only patients in the treatment effective group were found to have more PD-L1-positive patients(*P*<0.05), and each lymphocyte subpopulation, gender, age, BMI, smoking history, drinking history and extensive metastasis did not differ significantly between 2 groups of patients with dMMR. To exclude the effect of chemotherapy on immunotherapy, we divided the patients into the immunotherapy combined chemotherapy group (*n* = 173) and the immunotherapy without chemotherapy group (*n* = 22). Univariate analysis showed that CD4 + T cells were higher in the immunotherapy without chemotherapy group (*P* < 0.05) and CD8 + Tcells (*P* < 0.05) were lower in the effective group. The remaining MMR status, PD-L1 status, remaining lymphocyte subsets, gender, age, BMI, smoking history, drinking history and extensive metastasis did not differ between the two groups. Logistic analysis of CD4 + T cells, CD8 + T cells showed no statistical significance for all three. Univariate analysis showed that in the immunotherapy combined chemotherapy group CD4 + T cells, NK cells, and PD-L1-positive patients (*P* < 0.05) were higher in the effective group, and CD8 + T cells (*P* < 0.05) were lower. Inclusion of CD4 + T cells, PD-L1 status, and CD8 + T cells in the logistic analysis showed that PD-L1 (OR: 0.008, 95% CI: 0.001–0.065, *P* < 0.001), CD4 + T cells (OR: 0.911, 95% CI: 0.838–0.989, *P* = 0.043), NK cells (OR: 0.895, 95% CI: 0.831–0.966, *P* = 0.004) were factors affecting the short-term outcome of patients.

### Correlation of lymphocyte subsets with patient PFS

**Fig. 3:**
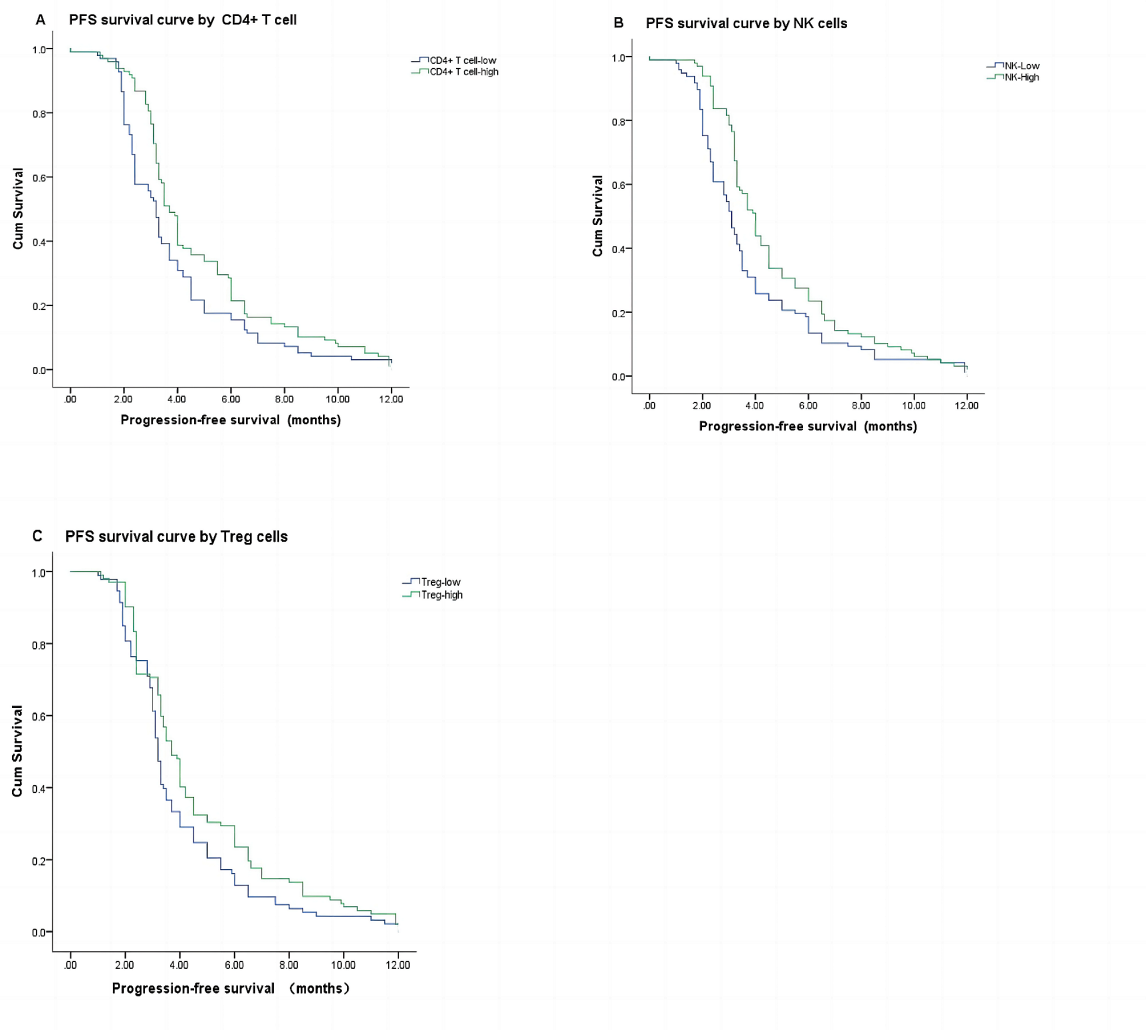
Kaplan–Meier survival curve of the correlation between the proportion of CD4 + T cell, NK cell、Treg and PFS

The median PFS in this study was 3.40 months. The median baseline CD4 + T cells, CD8 + T cells, CD4 / CD8 ratio, NK cells, Treg cells and B cells were used as cut-off values to divide the patients into high and low level groups. The median proportion of CD4 + T cell was 35.30%, CD8 + T cell was 30.90%, NK cell was 20.00%, Treg cell was 6.20%, the ratio of CD4+/CD8 + was 1.21. Univariate analysis found no significant correlation between age, BMI, smoking history, drinking history, CD8 + T cells, B cells and PFS. Extensive metastasis, gender, PD-L1 levels, MMR status, CD4 + T cells, the ratio of CD4 +/CD8+, NK cells, and Treg cells were associated with PFS (Table 4). The inclusion of extensive metastasis, gender, PD-L1 level, MMR status, CD4 + T cells, NK cells, and Treg cells in the Cox regression analysis showed that CD4 + T cells (HR = 0.950, 95% CI = 0.929–0.971, *P*<0.001), NK cells (HR = 0.951, 95% CI = 0.934–0.969 *P* < 0.001), Treg cells (HR = 0.816, 95% CI = 0.758–0.879, *P*<0.001), sex (HR = 1.416, 95% CI = 1.003-2.000, *P* = 0.048), MMR status (HR = 0.308, 95% CI = 0.141–0.674, *P* = 0.003), and PD-L1 level (HR = 0.448, 95% CI = 0.324–0.620, *P*<0.001) were independent prognostic predictors of PFS. Figure 3 shows the Kaplan-Meier curves for PFS in patients in the high level group and the low level group (CD4 + T cells, NK cells and Treg cells).

In addition we analyzed patients based on MMR status, treatment regimen. In the pMMR group, univariate analysis showed whether extensive metastasis (*P* = 0.032), PD-L1 level (*P* = 0.046), CD4 + T cells (*P* = 0.009), the ratio of CD4+/CD8+ (*P* = 0.001),CD8 + T cells (*P* = 0.030), NK cells (*P* = 0.003), Treg cells (*P* = 0.025) were associated with patient PFS. Whether patients had extensive metastasis, PD-L1 level, CD4 + T cells, CD8 + T cells, NK cells, and Treg cells in pMMR group were included in cox regression analysis. The results showed that CD4 + T cell (HR = 0.950, 95% CI = 0.921–0.979, *P* < 0.001), NK cell (HR = 0.957, 95% CI = 0.932–0.984 *P* = 0.002), Treg cell (HR = 0.826, 95% CI = 0.765–0.871, *P* < 0.001), PD-L1 level (HR = 0.463, 95% CI = 0.332–0.646, *P* < 0.001). In the dMMR group, univariate analysis showed that PD-L1 level affected the PFS of patients (*P* = 0.003), and gender, drinking history, smoking history, age, BMI, extensive metastasis, and lymphocyte subsets did not affect the PFS of patients. We divided the patients into the immunotherapy combined chemotherapy group (*n* = 173) and the immunotherapy without chemotherapy group (*n* = 22). Univariate analysis showed that MMR status, PD-L1 status, gender, extensive metastasis, the ratio of CD4+/CD8+,CD4 + T cell, and NK cell (*P* < 0.005) were associated with PFS in the immunotherapy combined chemotherapy group. The MMR status, PD-L1 status, gender, extensive metastasis, CD4 + T cells, and NK cell of patients in the immunotherapy combined chemotherapy group were included in the cox regression analysis. The results showed that CD4 + T cell (HR = 0.962, 95% CI = 0.939–0.984, *P* = 0.001), NK cell (HR = 0.961, 95% CI = 0.942–0.980 *P* < 0.001), MMR status (HR = 0.264, 95% CI = 0.114–0.615, *P* = 0.002), PD-L1 level (HR = 1,513, 95% CI = 1.087–2.108, *P* = 0.014) were factors influencing PFS. Univariate analysis showed that drinking history, MMR status, CD4 + T cell, NK-cell, and B cell (*P* < 0.005) were associated with patient PFS in the immunotherapy without chemotherapy group. Drinking history, MMR status, CD4 + T cell, NK cell, and B cell were included in cox regression analysis in the immunotherapy without chemotherapy group. The results showed that CD4 + T cell (HR = 0.910, 95% CI = 0.844–0.982, *P* = 0.014), MMR status (HR = 42.259, 95% CI = 1.874-952.917, *P* = 0.019), drinking history (HR = 10.455, 95% CI = 1.309–83.481, *P* = 0.027) were factors influencing PFS.

**Table 4:** Univariate analysis of the correlation between baseline lymphocyte subsets and PFS

	PFS[Month, (95%CI)]	Log rank	*P*
Age		0.109	0.741
<65	3.30(3.15–3.45)		
≥ 65	3.70(3.30–4.10)		
Gender		3.957	0.047
Female	3.30(2.99–3.61)		
Male	3.40(3.15–3.65)		
BMI		0.000	0.997
BMI<18.5kg/m2	3.30(3.09–3.53)		
BMI ≥ 18.5kg/m2	3.50(3.24–3.76)		
Smoking history		1.362	0.243
No	3.30(3.08–3.52)		
Yes	3.70(3.20–4.20)		
Drinking history		0.611	0.435
No	3.30(3.11–3.49)		
Yes	3.70(2.78–4.63)		
Extensive metastasis		4.679	0.031
No	3.70(3.18–4.22)		
Yes	3.30(3.15–3.45)		
PD-L1 expression		9.787	0.002
CPS<5	3.30(2.65–3.75)		
CPS ≥ 5	3.50(3.19–3.81)		
Mismatch repair status		9.068	0.003
pMMR	3.30(3.23–3.57)		
dMMR	11.00(0.00-22.92)		
CD4 + T		4.545	0.033
Low proportion group (<35.30%)	3.20(2.92–3.48)		
High proportion group(≥35.30%)	3.70(3.39–4.01)		
CD8 + T		2.689	0.101
Low proportion group (<30.90%)	3.70(3.22–4.18)		
High proportion group(≥30.90%)	3.30(3.11–3.49)		
CD4+/CD8+		5.152	0.023
Low ratio group (<1.21)	3.30(3.11–3.49)		
High ratio group (≥1.21)	3.50(2.93–4.07)		
NK		7.383	0.007
Low proportion group (<20.00%)	3.10(2.83–3.38)		
High proportion group(≥20.00%)	4.00(3.63–4.37)		
Tregs		4.058	0.044
Low proportion group (<6.20%)	3.20(3.05–3.35)		
High proportion group(≥6.20%)	3.70(3.35–4.05)		
B		0.762	0.383
Low proportion group (<5.30%)	3.30(3.07–3.53)		
High proportion group(≥5.30%)	3.50(3.20–3.80)		

## Discussion

Immunotherapy and its combination therapy are now frequently employed in the palliative care of advanced gastric cancer owing to the advancement of studies such as Keynote059 [8]. Since PD-L1 expression level, MMR status, and other biomarkers that are recommended by the guidelines are invasive and cannot be tracked dynamically, new blood biomarkers such circulating tumor cells and neutrophil-to-lymphocyte ratio (NLR) have been identified. Most studies have focused on tumor infiltrating immune cells (TIL), but immunotherapy is generally administered intravenously and has an impact on systemic immunity, and the T cell phenotype found in tumor tissue can usually also be detected in peripheral blood [9], thus peripheral blood lymphocyte subsets may be relevant to patient outcomes [10]. Studies have shown that patients with higher CD4 + T cells, CD8 + cells, and CD4+/CD8 + ratio in gastric cancer tumor tissue also have higher peripheral blood CD4 + T cells, CD8 + cells, and CD4+/CD8 + ratio [11]. The major goal of this study was to compare the changes in peripheral blood lymphocyte subsets before and after immunotherapy to determine whether they can predict the effectiveness and prognosis of immunotherapy in patients with advanced gastric cancer. We found that the proportions of CD4 + T cells, NK cells and Treg cells, gender, PD-L1 expression levels, and MMR status were independent prognostic indicators of PFS. We also showed that CD4 + T cell and NK cell could predict short-term patient outcomes and that the combination of CD4 + T cell and NK cell with PD-L1 expression levels was more effective than CD4 + T cells and NK cells alone.

In our study, the proportion of NK cells was found to be lower in patients under the age of 65, the proportion of CD8 + T cells was higher in PD-L1-negative patients than in PD-L1-positive patients, and the CD4+/CD8 + ratio was lower PD-L1-negative patients than that in PD-L1-positive patients in a study of the relationship between peripheral blood lymphocyte subsets and the clinicopathological characteristics of patients. The variation in NK cells between age groups may be due to the slow growth of tumors and limited inhibition immune function in elderly individuals. As with the previous Checkmate 649 study [12], this study confirmed a higher benefit of immunotherapy in patients with PD-L1 CPS ≥ 5. Our study also found that female sex were a risk factor for immunotherapy in gastric cancer, with reduced PFS in female patients compared to male patients. This is consistent with previous meta-analyses that men benefited more from immunotherapy than women in solid tumors [13], possibly due to the ability of androgens to promote recovery of CD8 + T cell function [14].

CD4 + T cells are necessary for CD8 + T cell activation, and ICI treatment requires a systemic CD4 + T cell response to obtain an effective CD8 + T cell response [15]. This study demonstrated that the proportion of CD4 + T cells increased in the effective group after immunotherapy. There was no significant change in the lymphocyte subsets in the effective group. This is consistent with Liu’s study, in which effective treatment elevated peripheral blood CD4 + T cells [16]. This suggests that effective immunotherapy may alleviate the body’s immunosuppressed state to some extent, promote the activation of immune cells and enhance the body’s anti-tumor effects. Immune function is not effectively restored in patients with a poor response to treatment. The proliferation of peripheral T cells after PD-1/PDL1 inhibitor treatment can promote more peripheral T cells to enter the tumor microenvironment, increase the number of tumor-infiltrating T cells and improve tumor immune infiltration, possibly transforming “cold tumors” into “hot tumors“ [17]. Previous studies have shown that higher peripheral CD4 + T cells were associated with smaller tumor size [18]. Changes in CD4 + T cells after immunotherapy may be a way to identify pseudo-progression after PD-1/PD-L1 inhibitor therapy. Our study showed that the CD4 + T cell was an independent predictor of both immunotherapy efficacy and prognosis in advanced gastric cancer, suggesting the indispensable tumor suppressive capacity of CD4 + T cells in immunotherapy. You et al. showed that low peripheral blood CD4 + T-cell levels in gastric cancer patients were associated with poorer overall survival [19]. Patients with higher peripheral blood CD4 + T cells before ICI treatment in non-small cell lung cancer [20]and colorectal cancer [21]have a better prognosis. This study also confirmed that the CD4+/CD8 + ratio was elevated in the effective group, which further emphasizes the importance of peripheral CD4 + T cells in the efficacy of ICI treatment. The elevated CD4+/CD8 + ratio also reflects an increase in the overall immune level of patients after effective PD-1/PD-L1 treatment [22].

CD8 + T cells are the main anti-tumor cells activated by PD-1/PD-L1 therapy [23]. The peripheral blood CD8 + T cells of patients in the effective group decreased mildly, which was the same as the changes observed in oral cancer and non-small cell lung cancer treated with PD-1 inhibitors in previous studies [24, 25]. However, it is inconsistent with previous results suggesting a burst of CD8 + T cell proliferation after PD-1 inhibitor treatment [26]. Possible reasons for this phenomenon are the following 2 points. First, previous studies have shown that most of the elevated peripheral CD8 + T cells after ICI treatment are caused by the proliferation of PD-1 + CD8 + T cells and that anti-PD-1 treatment releases CD8 + T cells previously suppressed by the PD-1/PD-L1 pathway. However, PD-1 expression is regulated by antigen load and, as tumor load decreases, tumor-specific CD8 + T cells may no longer express PD-1 [27], resulting in no proliferation or even a mild decline in peripheral CD8 + T cells after effective treatment. Second, proliferation of effective CD8 + T cells with ICI treatment occurs mostly at a very early stage. Previous studies have shown no PR or CR in patients whose proliferation of peripheral CD8 + T cells appeared later than six weeks after ICI treatment, and the late peripheral CD8 + T cell changes may not be related to PD-1 treatment [27].

Treg cells are part of CD4 + T cells (approximately 5%) [28] and patients with gastric cancer show increased numbers of regulatory T cells in the peripheral blood and tumor-infiltrating lymphocytes [29], however, the role of Treg cells in tumor progression remains controversial. High Treg cell infiltration is generally considered to be a poor prognostic factor [30]. However higher levels of tumor-infiltrating Treg cells in head and neck cancer [31], colorectal cancer [32] and melanoma [33] are associated with a better prognosis. The function of Treg cells has also varied in previous studies of gastric cancer.Li et al. showed that gastric cancer patients with high Treg cell infiltration had lower survival rates and more advanced TNM stages [18]. In contrast, Kim et al. showed that a higher density of Treg cells in the tumor microenvironment was associated with a good prognosis in patients with MSI-H gastric cancer [34]. The reason for the differential prognosis of Treg cells may be that immunosuppressive Treg cells may acquire an effector phenotype and contribute to anti-tumor immunity under certain conditions [35]. In the future, precise typing of Treg cells would be helpful in predicting the response to immunotherapy in patients with gastric cancer.

The anti tumor activity of NK cells is one of the bases for the efficacy of PD-1 /PD-L1 inhibitors [36]. NK cells also express PD-1, and the PD-1/PD-L1 pathway inhibits the cytotoxic potency of NK cells by approximately 10% [37], and anti-PD-1 treatment activates NK cells against tumors [38]. This is consistent with our study, where peripheral blood NK cells were increased in patients after effective anti-PD-1 treatment. Our study also demonstrated that peripheral blood NK cells were an independent predictor of the efficacy and prognosis of ICI therapy in patients with advanced gastric cancer. This is consistent with previous studies that anti-PD-1 therapy efficacy decreases after NK cell depletion [39]. A growing number of studies have highlighted that NK cells mobilization can influence the efficacy of PD-1/PD-L1 inhibitors [37]. The role of NK cells in anti-PD-1 therapy requires further investigation.

The functions of B cells in tumor growth and anti-PD-1 therapy are complex and different B cells may have different functions. CD20 + B cells are associated with a better prognosis in gastric cancer [40]. However, regulatory B cells may have anti-inflammatory properties and promote tumor growth [41]. Studies have shown that higher levels of tumor-infiltrating B cells are associated with better immunotherapeutic outcomes [42, 43]. Peripheral blood memory B cells can predict the efficacy of PD-1 inhibitors in non-small cell lung cancer to some extent [44]. In this study, peripheral blood B cells decreased after immunotherapy and did not correlate with the efficacy and prognosis of ICI treatment in advanced gastric cancer, probably because of the failure to differentiate among them. Further typing may be needed to investigate the correlation between peripheral B cells and the immunotherapy efficacy.

A subgroup analysis was also performed to analyze patients according to MMR status and whether immunotherapy was combined with chemotherapy. In the short-term efficacy analysis, PD-L1 status, CD4 + T cell, and NK cell were predictors of short-term efficacy in the pMMR group. dMMR group did not see lymphocyte subsets as predictors in short-term efficacy. In the immunotherapy plus chemotherapy group, PD-L1 status, CD4 + T cell, and CD8 + T cell were predictors of short-term efficacy. In the immunotherapy uncombined chemo group, CD4 + T cell was higher in the treatment-responsive group and CD8 + T cell was lower in the univariate analysisr analysis, but no statistically significant predictors of short-term efficacy were seen in the multivariate analysis. In the long-term outcome analysis, we found that CD4 + T cell, NK cell, Treg cell, and PD-L1 level were predictive of long-term outcome in patients with pMMR. Lymphocyte subsets were not predictive of long-term outcome in the dMMR group. CD4 + T cell, NK cell, MMR status, PD-L1 level were factors for PFS of patients in the immunotherapy plus chemotherapy group. CD4 + T cells, MMR status, and drinking history were factors for PFS of patients in the immunotherapy without chemotherapy group. The above results showed that lymphocyte subsets were predictive in all subgroups except for dMMR group, but due to the limited sample size, we still believe that more data are needed to support the predictive effect of different lymphocyte subsets in dMMR patients and different immunotherapy regimens.

The limitations of our study include its retrospective design, which made it difficult to control for confounding factors; its focus on patients’ dMMR status rather than their MSI status, which raises the possibility that some dMMR patients still are MSI-L or MSS; its lack of additional molecular typing of peripheral blood lymphocyte subsets, which may be a limitation; and its failure to collect absolute counts of lymphocyte subsets.

## Conclusions

In this study, successful PD-1 inhibitor therapy caused modifications in specific subsets of peripheral blood lymphocytes. In patients with advanced gastric cancer, high proportions of CD4 + T cells and NK cells were independent predictors of the short-term efficacy of immunotherapy, and the predictive value of combined CD4 + T cell, NK cell, and PD-L1 expression level is stronger than that of individual CD4 + T cells or NK cells. In patients with advanced gastric cancer, a high proportion of CD4 + T cells, NK cells, and Treg cells were independent predictors of PFS with immunotherapy. Peripheral blood lymphocyte subsets are promising biomarkers in clinical applications of PD-1 inhibitors in patients with advanced gastric cancer.

### Electronic supplementary material

Below is the link to the electronic supplementary material.

Supplementary Material 1

Supplementary Material 2

Supplementary Material 3

## Data Availability

The datasets generated during the current study are not publicly available due to ethical restrictions, but are available from the corresponding author on reasonable request.

## Abbreviations

ICI: Immune checkpoint inhibitor
PFS: Progression-free survival
Tregs: Regulatory T cells
PD-L1: Programmed cell death ligand 1
TILs: Tumor-infltrating lymphocytes
BMI: Body Mass Index
MMR: Mismatch repair status
dMMR: mismatch repair deficient
pMMR: mismatch repair proficient
CT: Computed tomography
MRI: Magnetic resonance imaging
PET/CT: positron emission tomography/computed tomography
CR: Complete response
PR: Partial response
SD: Stable disease
PD: Disease progression
RECIST: The solid tumor response evaluation criteria
