# Supplementary material for: Association of lymphocyte subsets with the efficacy and prognosis of PD‑1 inhibitor therapy in advanced gastric cancer: results from a monocentric retrospective study
Source: BMC Gastroenterol. 2024 Mar 15;24:113. doi: 10.1186/s12876-024-03168-0 (PMC10943815; doi:10.1186/s12876-024-03168-0)
Supplement: Supplementary file 1 — Supplementary Material 1 [file 12876_2024_3168_MOESM1_ESM.docx]

**PD‑L1 immunohistochemistry assessment**

**Materials** anti-human PD-L1 antibody (DAKO 22C3), manufacturer Agilent (USA), monoclonal antibody clone number (22C3), monoclonal antibody dilution ratio 1:50.

**Methods** Immunohistochemical staining was performed on the VENTANA BenchMark Ultra platform using the kit accompanying the instrument. PD-L1 positive immunoreactive staining was localized to the cell membrane and assessed using the combined positive score (CPS), which is 100 times of the ratio of the total number of immunoreactively stained tumor cells, lymphocytes and macrophages in the 20x objective field to the total number of tumor cells in the field.
