# Supplementary material for: Association of lymphocyte subsets with the efficacy and prognosis of PD‑1 inhibitor therapy in advanced gastric cancer: results from a monocentric retrospective study
Source: BMC Gastroenterol. 2024 Mar 15;24:113. doi: 10.1186/s12876-024-03168-0 (PMC10943815; doi:10.1186/s12876-024-03168-0)
Supplement: Supplementary file 2 — Supplementary Material 2 [file 12876_2024_3168_MOESM2_ESM.docx]

**Mismatch repair status immunohistochemistry assessment**

**Materials** MSH6 monoclonal antibody, clone number (EP49), Beijing ZhongShan Jinqiao Biotechnology Co., Ltd, dilution ratio 1:100; MSH2 monoclonal antibody, clone number (RED2), Beijing ZhongShan Jinqiao Biotechnology Co. Ltd. at a dilution ratio of 1:5000; MLH1 monoclonal, antibody clone number (GM002), Beijing ZhongShan Jinqiao Biotechnology Co. Ltd. at a dilution ratio of 1:500; PMS2 monoclonal, antibody clone number (EP51), GeneScience Co. at a dilution ratio of 1:70.

**Methods** MMR staining was performed by immunohistochemical staining on the Leica BOND III platform using the platform's kit. Absence of any one or more of the four MMR proteins (MLH1, MSH2, MSH6 and PMS2) was defined as mismatch repair deficient (dMMR), and all positives were defined as mismatch repair proficient (pMMR)
