# Supplementary material for: Association of lymphocyte subsets with the efficacy and prognosis of PD‑1 inhibitor therapy in advanced gastric cancer: results from a monocentric retrospective study
Source: BMC Gastroenterol. 2024 Mar 15;24:113. doi: 10.1186/s12876-024-03168-0 (PMC10943815; doi:10.1186/s12876-024-03168-0)
Supplement: Supplementary file 3 — Supplementary Material 3 [file 12876_2024_3168_MOESM3_ESM.docx]

**Blood collection and fow cytometry**

**Materials** All following materials’’ company is Beijing Tongsheng Shidai Biotechnology Co.. CD4+ T cells, CD8+ T cells: CD45-PerCP-Cy5-5, product number Z6410002; CD3-FITC, product number Z6410002; CD4-APC, product number Z6410002; CD8-PE, product number Z6410002. NK cells cells: CD16+56-PE, product number Z6410010; CD45-PerCP-Cy5.5 , product number Z6410010; CD3-FITC , product number Z6410010; CD19-APC, product number Z6410010. Tregs: CD45-PerCP-Cy5.5, product number Z6410010; CD4-FITC, product number Z6410005; CD25-APC, product number Z6410045-100T; CD127-PE, product number Z6410046-100T.

**METHODS** The proportion of lymphocyte subsets in the peripheral blood was measured using a BD FAS-Canto Ⅱ flow cytometer and flow antibodies. Three flow cytometry tubes were prepared for each peripheral blood specimen, and labelled Tube 1, Tube 2, Tube 3. CD45-PerCP-Cy5.5, CD3-FITC, CD4-APC and CD8-PE were added to tube 1 to detect the proportion of CD4+ T cells (CD3+CD4+) and CD8+ T cells (CD3+CD8+) in peripheral blood lymphocytes. In tube 2 CD16+56-PE, CD45-PerCP-Cy5.5, CD3-FITC and CD19-AP were added to detect the proportion of NK cells (CD45+CD3-CD16+CD56+) and B cells (CD45+CD3-CD19+) in peripheral blood lymphocytes. CD45-PerCPCy5.5, CD4-FITC, CD25-APC and CD127-PE were added to tube 3 to detect the proportion of Treg cells (CD45+CD4+CD25HICD127LOW) in peripheral blood lymphocytes. 100μl of anticoagulated peripheral blood was added to each tube and incubated for 20-30 minutes at room temperature. After staining, 1mL of erythrocyte lysate was added to each tube. Shake and leave in the dark at room temperature for 10 minutes and centrifuge at 300g for 5 minutes. The supernatant was discarded, 1 ml of PBS was added to each tube, washed once and 50 μl of PBS was added to each tube and the cell subpopulation ratios were examined by flow cytometry. Finally the data was analyzed using MULTISET software. The CD4+/CD8+ ratio was calculated by dividing the proportion of CD4+ T cells by the proportion of CD8+ T cells. According to the flow cytometry testing platform at the First Hospital of Shanxi Medical University, the normal reference ranges for the above parameters are: CD4+ T cells (30-50%), CD8+ T cells (20-35%), CD4+/CD8+ ratio (1-2), NK cells (20-35%), Treg cells (3-7%) and B cells (5.6-16%).
